# The role of microbiome dysbiosis in cardiovascular disease: Mechanisms and therapeutic implications

**DOI:** 10.21542/gcsp.2025.3

**Published:** 2025-02-28

**Authors:** Razan Abdulaal, Yehya Tlaiss, Fatima Jammal, Tayib Hadi Moussbah, Alaa Tarchichi, Ali Hteit, Mohamad Tlais, Dana Nassif

**Affiliations:** 1 Faculty of Medicine and Medical Sciences, University of Balamand, Beirut, Lebanon; 2 Department of Nutrition and Food Sciences, American University of Beirut, Beirut, Lebanon

## Abstract

The gut microbiome plays a critical role in cardiovascular disease (CVD) pathogenesis through systemic inflammation, disrupted lipid metabolism, and proatherogenic metabolites like trimethylamine-N-oxide (TMAO). Dysbiosis contributes to increased intestinal permeability, platelet hyperreactivity, and reduced short-chain fatty acids (SCFAs), exacerbating cardiovascular risk. Emerging microbiome-targeted therapies, including probiotics, prebiotics, fecal microbiota transplantation (FMT), and dietary interventions, show promise in mitigating CVD. However, challenges remain in translating these findings into clinical practice due to strain-specific effects and interindividual variability. The gut-heart axis represents a transformative avenue for CVD prevention and management, warranting further research to optimize long-term efficacy and safety.

## Introduction

The human gastrointestinal (GI) tract is one of the body’s most extensive interfaces, spanning 250–400 m^2^, where it interacts with a range of environmental factors and antigens^1^. The microbiome encompasses the complete genetic repertoire of all microorganisms within a given environment. This includes not only the microbial community but also its structural components, metabolic byproducts, and the environmental conditions that shape its composition and function^2^. This community has co-evolved with its human host over millennia, forming a complex, mutually beneficial relationship. The gut microbiota is estimated to contain up to 10^1^^4^ microorganisms^3^, and more than 100 times the genetic material of the human genome^4^. However, recent studies have refined this view, proposing that human and bacterial cells may exist in nearly equal numbers, with a ratio close to 1:1^5^.

While the terms “microbiota” and “microbiome” are often used interchangeably, they have distinct meanings. Microbiota refers specifically to the community of living microorganisms residing in a particular environment, such as the gut or oral cavity. In contrast, the microbiome encompasses the collective genetic material of these microorganisms, along with their structural components, metabolites, and the environmental factors influencing their activity. Thus, the microbiome represents a broader and more comprehensive concept than microbiota. This review focuses primarily on the roles of microbiota in maintaining human health and contributing to disease^2^.

The gut microbiome contributes to human health through a critical function known as colonization resistance, whereby a diverse microbial community mitigates the risk of local colonization with potentially pathogenic bacteria^6^. This is achieved through several mechanisms including competing with pathogens for essential nutrients and physical space and secreting bacteriocins and other antimicrobial compounds that induce pore formation, nucleic acid degradation, and/or interference with cell wall synthesis^7^.

The gut microbiome affects immune health by modulating both innate and adaptive immunity. Commensal bacteria interact with the host’s immune system to help distinguish between harmful pathogens and beneficial microbes, which helps regulate inflammation^6^. For instance, gut microbiota can stimulate the secretion of immunoglobulin A and promote a balance between immune-stimulating T helper cells and immune-suppressing regulatory T cells^6,8^. Additionally, specific bacteria, such as *Faecalibacterium prausnitzii*, play a role in decreasing pro-inflammatory cytokines (e.g., IL-12) while enhancing anti-inflammatory cytokines (e.g., IL-10)^8^.

Gut microbiota also ferment unabsorbed starches and dietary fiber, producing short-chain fatty acids (SCFAs) such as butyrate, propionate, and acetate, which play roles beyond energy production, including regulating appetite, enhancing glucose tolerance, and modulating immune responses^9^.

The gut microbiota are crucial for synthesizing essential vitamins, such as B vitamins (e.g., cyanocobalamin) and vitamin K, which support vital metabolic functions, with shifts in microbial populations potentially impacting vitamin availability^8^.

Microbiome dysbiosis refers to a disruption in the gut microbiome that deviates from its balanced, healthy state, known as eubiosis^10^. Dysbiosis is marked by changes in both the composition and function of the microbiome, often involving a loss of microbial diversity and a shift in the balance between beneficial and potentially harmful microbial populations within a disease state or phenotype^11^. This altered microbial landscape has wide-reaching implications for systemic health, as it can promote inflammatory and metabolic disruptions that contribute to the onset and progression of diseases, particularly cardiovascular disease (CVD).

Numerous studies have identified associations between cardiovascular disease (CVD) phenotypes and alterations in specific microbial taxa, as well as changes in gut bacterial richness and diversity. Initial research exploring a causal relationship between the gut microbiome and CVD highlighted the role of trimethylamine N-oxide (TMAO), a metabolite produced through the breakdown of dietary nutrients prevalent in Western diets, such as lecithin, choline, and carnitine^12^. Early studies found bacterial DNA within atherosclerotic plaques, with microbial signatures linked to disease-associated taxa^13^. Additionally, shifts in microbial composition have been observed in patients with various CVD risk factors, including hypertension, dyslipidemia, insulin resistance, and other metabolic abnormalities, suggesting that gut microbiome alterations may be a significant factor in CVD risk^14^.

The human body functions through an intricate network of interdependent systems, each playing a vital role in maintaining health and well-being. Recent advancements in medical research have highlighted the profound connections between these systems, with the emerging field of the “gut-heart axis” gaining significant attention for its impact on cardiovascular health. The gut-heart axis refers to the complex, bidirectional connection between the gut microbiome and cardiovascular health, mediated through interactions involving the immune system, metabolic pathways, and physiological factors. This axis highlights how the gut microbiota can influence heart function and disease development through the production of metabolites, modulation of inflammatory pathways, and impacts on systemic health^15^. For example, gut bacteria can produce pro-inflammatory molecules such as trimethylamine-N-oxide (TMAO), derived from dietary components like choline and carnitine, which are linked to arterial plaque formation and increased cardiovascular risk^15^.

Disruptions in the gut microbiome, or dysbiosis, can trigger systemic inflammation through pathways such as NOD1, NOD2, and NLRP3 inflammasome activation, which are central to the development of cardiovascular diseases (CVD)^15^. Additionally, gut microbial byproducts like short-chain fatty acids (SCFAs) can exert protective effects by reducing inflammation^15^, demonstrating the dual role of the gut microbiota in promoting or mitigating heart disease.

**Figure 1.**
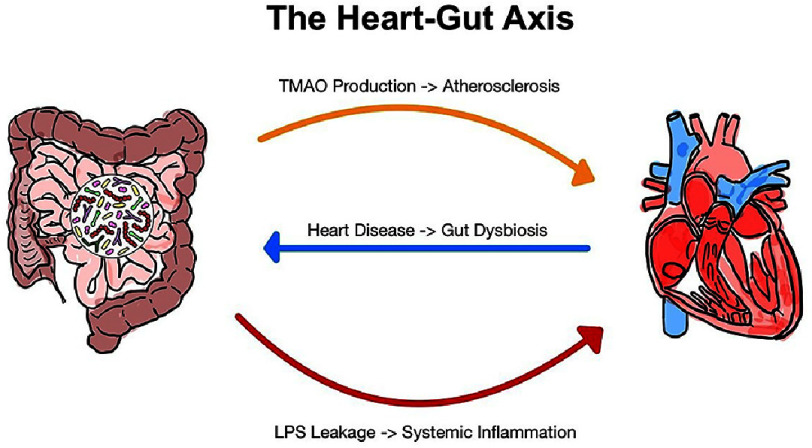
The heart-gut axis shwoing bidirectional interactions between the gut microbiome and cardiovascular health.

The gut-heart axis is shaped by a multifaceted interplay between diet, environmental factors, microbiota composition, and host physiology (Figure 1)^15^. Its growing recognition underscores the need for multidisciplinary research to understand the molecular and cellular mechanisms that govern this relationship. Maintaining a healthy gut-heart axis is essential for preventing CVD and other systemic diseases, making it a promising target for novel therapeutic approaches.

This review aims to provide an in-depth exploration of the mechanisms linking gut microbiome dysbiosis to cardiovascular disease (CVD), drawing on the latest advancements in microbiology, cardiology, and immunology. By examining the intricate interplay between microbial imbalances, inflammatory pathways, and metabolic processes, this review seeks to shed light on how dysbiosis contributes to CVD pathogenesis. Additionally, it evaluates emerging microbiome-targeted therapies, highlighting their potential to transform the prevention and treatment of CVD.

## Methods

We conducted a comprehensive literature review using the PubMed database to identify relevant studies on microbiome dysbiosis and cardiovascular disease (CVD). The search was performed using key Medical Subject Heading (MeSH) terms, including “gut microbiome,” “cardiovascular disease,” “TMAO,” “dysbiosis,” “inflammation,” and “gut-heart axis.”

Studies were included if they were published in peer-reviewed journals, available in English, investigated the mechanisms linking gut microbiome dysbiosis to CVD or evaluated microbiome-targeted interventions, and consisted of original research articles, systematic reviews, or meta-analyses. Exclusion criteria included studies that lacked primary data, such as editorials, commentaries, or case reports, those focused on microbiome research unrelated to CVD or systemic inflammation, and animal-only studies without translational relevance to human physiology.

The literature search covered studies published from January 2010 to November 2024 to ensure the inclusion of the latest advancements. A total of 178 studies were initially retrieved. After screening abstracts and full texts, 52 studies met the inclusion criteria and were included in the final analysis. To assess the methodological rigor of the included studies, we applied the Newcastle-Ottawa Scale (NOS) for observational studies and the Cochrane Risk of Bias (RoB 2) tool for randomized controlled trials (RCTs). Studies were categorized as low, moderate, or high quality, with only moderate-to-high quality studies included in the review. The selection process involved two independent reviewers screening the titles and abstracts of retrieved articles, with disagreements resolved through discussion or consultation with a third reviewer. Full-text articles were then reviewed to ensure they met the predefined inclusion criteria.

### Mechanisms linking microbiome dysbiosis to cardiovascular disease

#### TMAO production and atherosclerosis

Trimethylamine N-oxide (TMAO), a metabolite derived from gut microbiota, has emerged as a significant contributor to cardiovascular disease (CVD). The production of TMAO begins with the microbial metabolism of dietary precursors such as choline, phosphatidylcholine, and L-carnitine, which are abundant in red meat, eggs, and dairy products. These compounds are metabolized into trimethylamine (TMA) by gut bacterial enzymes, including the choline TMA lyase (CutC/D) and carnitine Rieske-type oxygenase/reductase systems (CntA/B and YeaW/X). TMA is absorbed into the portal circulation and oxidized into TMAO by flavin-containing monooxygenase-3 (FMO3) in the liver^16^. Elevated TMAO levels have been linked to endothelial dysfunction, impaired reverse cholesterol transport, and foam cell formation—key contributors to atherogenesis^17,18^. Each 10 µmol/L increase in plasma TMAO levels is associated with a 7.6% increased risk of major cardiovascular events, including myocardial infarction and stroke^12,16^. Another study found that patients in the highest quartile of TMAO levels had a 2.5-fold higher risk of cardiovascular mortality compared to those in the lowest quartile^18^.

Clinical studies have provided robust evidence linking circulating TMAO concentrations with increased risk of CVD. For example, individuals with elevated plasma TMAO levels have demonstrated higher rates of major adverse cardiovascular events, including myocardial infarction and stroke^15^. Moreover, prospective studies have revealed that patients in the highest quintile for plasma TMAO levels are at significantly higher risk of developing recurrent CVD events. Experimental models further support these findings; diets enriched with TMAO precursors accelerate vascular lesion formation by disrupting cholesterol clearance and promoting deposition within arterial walls^18^. Antibiotic interventions that reduce gut bacterial production of TMA have been shown to suppress TMAO levels and attenuate these effects, reinforcing the metabolite’s role in disease progression^19^.

**Figure 2.**
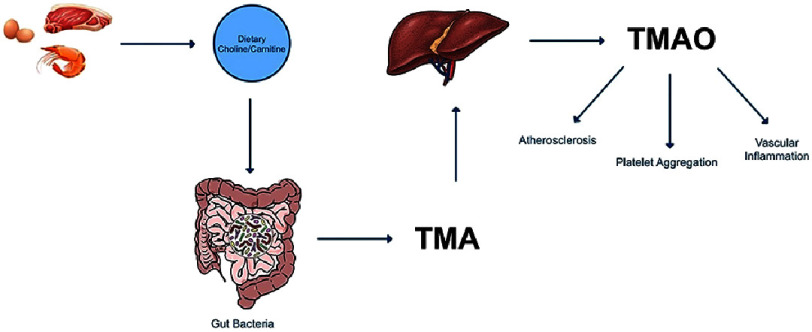
Pathway of TMAO production and its cardiovascular effects.

The mechanistic role of TMAO in atherogenesis is increasingly well understood. One key pathway involves its inhibition of bile acid synthesis, which disrupts cholesterol homeostasis and promotes vascular lipid accumulation^18^. TMAO also enhances inflammation by activating NLRP3 inflammasomes, leading to increased endothelial cell injury and recruitment of inflammatory mediators^16^. Another critical mechanism is its effect on platelet function. Elevated TMAO levels have been shown to increase platelet responsiveness to agonists, thereby amplifying the risk of thrombosis, a major contributor to cardiovascular complications^18^. (Figure 2) summarizes the TMAO pathway.

#### Gut-derived inflammation and cardiovascular risk

**Figure 3.**
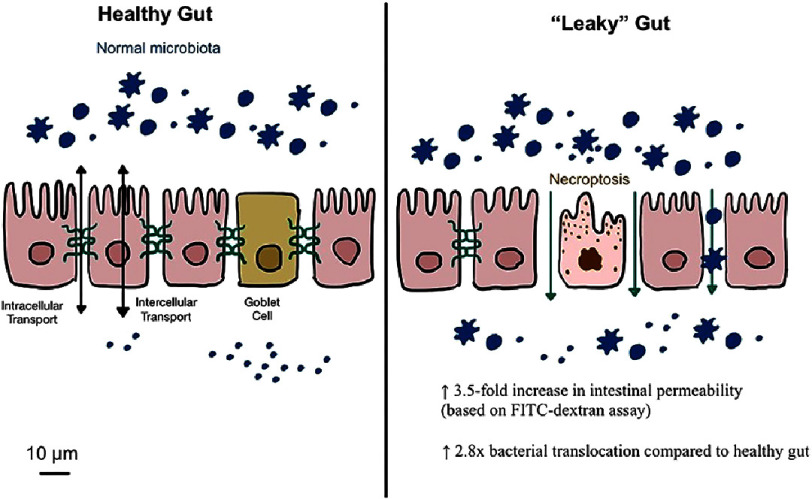
Comparison of healthy and leaky gut barrier integrity.

The gut microbiota significantly influences systemic inflammation, a key driver of cardiovascular disease (CVD). Disruption of the intestinal microbiota, commonly referred to as dysbiosis, alters the gut barrier and facilitates the translocation of microbial products into systemic circulation. One critical outcome of this disruption is increased intestinal permeability, often termed “leaky gut” (Figure 3), which allows endotoxins such as lipopolysaccharides (LPS) to escape into the bloodstream^20^. These endotoxins bind to Toll-like receptor 4 (TLR4) on immune cells, triggering pro-inflammatory cascades that contribute to systemic inflammation and endothelial dysfunction—key precursors of atherosclerosis and CVD^21^.

LPS-mediated endotoxemia is strongly associated with the progression of cardiovascular conditions. Elevated circulating LPS levels stimulate the production of pro-inflammatory cytokines, including interleukin-6 (IL-6), tumor necrosis factor-alpha (TNF-*α*), and interleukin-1 beta (IL-1*β*), which exacerbate endothelial injury and promote the recruitment of monocytes to vascular sites. These mechanisms establish a vicious cycle of chronic inflammation and vascular damage^22^. Experimental studies have demonstrated that animals exposed to high-fat diets, which induce dysbiosis and endotoxemia, exhibit accelerated atherosclerosis, highlighting the interplay between dietary habits, gut microbiota, and cardiovascular health^23^.

The intestinal barrier itself is a dynamic and multifaceted structure comprising epithelial cells, tight junction proteins, and a protective mucus layer. Dysbiosis disrupts this barrier by degrading tight junction proteins, such as zonulin and occludin, thereby increasing permeability. Loss of barrier integrity facilitates not only the translocation of LPS but also other microbial metabolites, such as peptidoglycans and flagellins, further amplifying systemic inflammation^21^. Studies have indicated that interventions targeting gut barrier restoration, such as the use of dietary fibers or probiotics, can mitigate inflammation and reduce CVD risk^23^.

Beyond LPS, other gut-derived factors, including microbial metabolites like short-chain fatty acids (SCFAs), influence cardiovascular health. SCFAs, produced through bacterial fermentation of dietary fibers, possess anti-inflammatory properties and help maintain the integrity of the gut barrier. However, dysbiosis often results in reduced SCFA production, depriving the host of their protective effects^22^. Restoration of SCFA levels has been proposed as a therapeutic strategy to modulate inflammation and improve cardiovascular outcomes.

In summary, gut-derived inflammation represents a critical link between intestinal health and cardiovascular risk. Dysbiosis, endotoxemia, and barrier dysfunction collectively exacerbate systemic inflammation and vascular damage, underscoring the need for strategies targeting gut health in the prevention and management of CVD^23,24^.

#### Short-chain fatty acids (SCFAs) and blood pressure regulation

Short-chain fatty acids (SCFAs), primarily acetate, propionate, and butyrate, are pivotal metabolites derived from gut microbiota fermentation of dietary fibers. These molecules have gained recognition for their profound impact on host physiology, including blood pressure (BP) regulation. The relationship between SCFAs and BP control underscores the significance of gut microbiota in cardiovascular health, mediated by signaling pathways and systemic interactions^25,26^.

SCFAs exert their effects through various mechanisms, including activation of G-protein-coupled receptors (GPCRs) such as GPR41, GPR43, and Olfr78, and inhibition of histone deacetylases (HDACs). Activation of GPR41 and GPR43 has been shown to regulate vascular tone and systemic inflammation, key contributors to BP control. For instance, GPR41 activation promotes vasodilation and improves nutrient delivery, whereas Olfr78, expressed in renal vasculature, modulates renin secretion, impacting BP homeostasis^26,27^. Additionally, SCFAs regulate immune responses by influencing regulatory T cells and reducing pro-inflammatory cytokines like interleukin-17 and tumor necrosis factor-alpha, thereby mitigating hypertensive damage^28^.

Clinical and experimental studies have highlighted the role of SCFAs in lowering BP. High-fiber diets, which enhance SCFA production, have been associated with reduced BP levels. Animal models of hypertension have shown that supplementation with propionate, a key SCFA, decreases BP and attenuates cardiac and vascular damage by promoting anti-inflammatory effects and maintaining endothelial integrity^25,28^. Conversely, dysbiosis and reduced SCFA production are linked to increased BP and systemic inflammation, as observed in germ-free animals and those on fiber-deficient diets^26,29^.

The interplay between SCFAs and the renin-angiotensin system (RAS) further emphasizes their role in BP regulation. By modulating renin secretion and vascular responsiveness, SCFAs directly influence hemodynamic stability. Moreover, their impact on the gut-brain axis and autonomic nervous system suggests a broader regulatory framework encompassing both central and peripheral mechanisms^30^.

In summary, SCFAs represent a critical link between diet, gut microbiota, and BP regulation. Strategies to enhance SCFA production through dietary interventions, prebiotics, and probiotics hold promise as non-pharmacological approaches to managing hypertension and reducing cardiovascular risk^25,27^.

#### Microbiota and lipid metabolism in CVD

The gut microbiota plays a critical role in lipid metabolism, influencing cardiovascular health through a complex interplay of microbial metabolites and host signaling pathways. Dysbiosis of the gut microbiome has been linked to disruptions in cholesterol metabolism, bile acid transformation, and lipid absorption, contributing to cardiovascular disease (CVD) pathogenesis^31,32^. The microbiota not only regulates lipid homeostasis but also impacts systemic lipid profiles and atherosclerosis progression.

One of the primary mechanisms involves the conversion of primary bile acids into secondary bile acids by gut bacteria. Secondary bile acids, such as deoxycholic acid, regulate cholesterol metabolism and absorption. Dysbiosis shifts the bile acid composition, reducing hydrophilic bile acids, which have cardioprotective effects, and increasing hydrophobic bile acids, which are associated with vascular inflammation and endothelial dysfunction^32^. This imbalance contributes to lipid deposition in arterial walls, accelerating atherosclerosis development^33^.

Cholesterol metabolism is another pathway influenced by gut microbiota. Certain bacterial strains, including members of the genera Lactobacillus and Bifidobacterium, express bile salt hydrolase enzymes that facilitate cholesterol conversion into coprostanol, a less absorbable form excreted in feces. This process reduces circulating cholesterol levels, mitigating a major risk factor for CVD^31^. Conversely, dysbiosis can enhance cholesterol absorption and hinder reverse cholesterol transport, further contributing to plaque formation in arteries^34^.

The microbiota also modulates systemic lipid profiles through short-chain fatty acids (SCFAs) and other bioactive metabolites. SCFAs, derived from fiber fermentation, influence lipid synthesis in the liver by activating G-protein-coupled receptors such as GPR41 and GPR43. This regulation reduces triglyceride accumulation and promotes high-density lipoprotein (HDL) synthesis, offering protection against CVD^33^. Dysbiosis, characterized by reduced SCFA production, is associated with hyperlipidemia and an increased risk of cardiovascular events^34^.

Emerging evidence suggests that therapeutic modulation of the gut microbiome can restore lipid balance and reduce cardiovascular risk. Strategies such as dietary interventions, probiotics, and prebiotics aim to enhance the abundance of beneficial bacteria that promote cholesterol excretion and bile acid recycling^31^. For example, supplementation with probiotics containing Lactobacillus strains has demonstrated efficacy in lowering LDL cholesterol levels and improving overall lipid profiles in clinical trials^33^.

In conclusion, the gut microbiota exerts profound effects on lipid metabolism through its influence on bile acid transformation, cholesterol absorption, and systemic lipid regulation. Dysbiosis disrupts these processes, contributing to the pathogenesis of CVD. Targeting the microbiota to restore lipid homeostasis represents a promising avenue for therapeutic intervention in cardiovascular health^31,32^.

### Therapeutic interventions targeting the gut microbiome to reduce cardiovascular risk

#### Probiotics and prebiotics

Probiotics and prebiotics have garnered significant attention for their potential to modulate the gut microbiota and mitigate cardiovascular disease (CVD) risk. Probiotics are live microorganisms that confer health benefits to the host when consumed in adequate amounts. They work through mechanisms including bile acid deconjugation, cholesterol assimilation, and the enhancement of short-chain fatty acid (SCFA) production. These SCFAs, particularly acetate, propionate, and butyrate, influence systemic inflammation and lipid metabolism by activating G-protein-coupled receptors (e.g., GPR41, GPR43) and inhibiting histone deacetylases, which regulate gene expression and immune responses^35,36^. Prebiotics, on the other hand, are non-digestible fibers that selectively stimulate the growth of beneficial gut bacteria such as *Bifidobacteria* and *Lactobacilli*. They enhance SCFA production and modulate the gut-liver axis, contributing to reduced systemic inflammation and improved lipid profiles^37,38^.

Probiotic strains such as *Lactobacillus acidophilus*, *Lactobacillus reuteri*, and *Bifidobacterium lactis* are particularly effective in reducing cholesterol levels by deconjugating bile salts. This leads to increased bile acid excretion and a compensatory uptake of circulating cholesterol for bile acid synthesis in the liver. Additionally, probiotics stabilize the gut barrier, reduce lipopolysaccharide (LPS) translocation, and decrease pro-inflammatory cytokines, thus lowering chronic inflammation associated with atherosclerosis and CVD^39^.

Numerous clinical trials and meta-analyses substantiate the cardiovascular benefits of probiotics and prebiotics. For example, *Lactobacillus reuteri NCIMB 30242* has been shown to reduce LDL cholesterol levels by up to 11% in hypercholesterolemic patients, comparable to standard therapeutic lifestyle changes^37^. Similarly, supplementation with synbiotics (a combination of probiotics and prebiotics) has demonstrated improvements in inflammatory markers, such as reductions in C-reactive protein (CRP) and tumor necrosis factor-alpha (TNF-*α*), as well as enhancements in antioxidant markers like glutathione and total antioxidant capacity^38^. *Lactobacillus acidophilus* and *Bifidobacterium lactis* combined with inulin lowered C-reactive protein (CRP) by 22%, reducing inflammation^38^.

A meta-analysis of randomized controlled trials revealed significant reductions in total cholesterol, LDL cholesterol, and triglycerides with probiotic use. The same analysis highlighted improvements in high-density lipoprotein (HDL) cholesterol levels, further supporting their role in lipid modulation and CVD risk reduction^36^. Prebiotics such as inulin and fructooligosaccharides synergize with probiotics to enhance SCFA production, improve gut barrier integrity, and modulate immune responses, providing additional cardiovascular benefits^38^.

Despite promising findings, several challenges hinder the widespread clinical adoption of probiotics and prebiotics for CVD prevention and treatment. One of the most pressing issues is the strain-specific nature of probiotic effects, which necessitates precise selection of strains and formulations tailored to individual patient profiles. Variability in response due to differences in baseline microbiota composition, age, genetics, and comorbidities further complicates their application^35^. Determining optimal dosages and administration regimens is also a challenge, as current studies use varying doses, ranging from 10^7^ to 10^13^ colony-forming units per day, with inconsistent outcomes^37^.

Another limitation is the lack of long-term safety data and the potential for adverse effects, particularly in immunocompromised patients. While probiotics are generally recognized as safe, their interactions with other medications and their impact on systemic health need further exploration^36^. Additionally, the quality and efficacy of commercially available probiotic and prebiotic supplements vary widely, with some products failing to deliver live microorganisms at effective concentrations^38^.

To fully harness the potential of probiotics and prebiotics in cardiovascular health, future research must address these limitations through well-designed, large-scale clinical trials. These trials should focus on identifying specific probiotic strains and prebiotic compounds that are most effective for CVD prevention and treatment, while also examining their long-term safety and efficacy. Advances in microbiome sequencing and personalized medicine could pave the way for tailored interventions based on individual microbiota profiles, optimizing outcomes and minimizing variability in response^40^.

Integrating probiotics and prebiotics into dietary recommendations and clinical practice could also enhance adherence and accessibility. Strategies such as fortifying functional foods with probiotics, educating healthcare providers about their benefits, and developing regulatory standards for supplement quality are crucial for their successful implementation^39^.

Probiotics and prebiotics represent a promising adjunctive approach for reducing cardiovascular risk through their modulation of the gut microbiota, regulation of lipid metabolism, and anti-inflammatory effects. While challenges remain, ongoing research and technological advancements in microbiome science offer hope for their integration into mainstream CVD management.

#### Dietary interventions

The role of dietary interventions in cardiovascular health is well established, with the Mediterranean diet (MedDiet) and plant-based diets emerging as particularly effective approaches to modulate the gut microbiota and reduce cardiovascular disease (CVD) risk. These diets, rich in fiber, polyphenols, and unsaturated fats, exert their effects by improving lipid metabolism, enhancing gut microbiota composition, and attenuating systemic inflammation^41,42^.

The Mediterranean diet, characterized by high consumption of fruits, vegetables, whole grains, legumes, nuts, and extra virgin olive oil, along with moderate intake of fish and poultry, has demonstrated significant benefits in cardiovascular prevention. A critical component of this diet is its high fiber content, which promotes the growth of beneficial gut bacteria, such as *Faecalibacterium prausnitzii* and *Bacteroides uniformis*. These microbial shifts are associated with increased production of short-chain fatty acids (SCFAs), which regulate vascular tone, lipid metabolism, and inflammatory pathways^43,44^.

Epidemiological studies and clinical trials underscore the efficacy of the Mediterranean diet in reducing cardiovascular events. For example, the PREDIMED trial showed that adherence to a Mediterranean diet enriched with extra virgin olive oil or nuts reduced the incidence of major cardiovascular events by approximately 30% compared to a low-fat diet. These benefits are attributed to the diet’s ability to improve endothelial function, reduce oxidative stress, and modulate the gut microbiome towards a more cardioprotective profile^40,41^. Similarly, plant-based diets, emphasizing high intake of vegetables and legumes while minimizing animal products, have been associated with reduced blood pressure and improved lipid profiles^43^.

Randomized controlled trials and meta-analyses provide robust evidence supporting the cardiovascular benefits of these dietary patterns. The MedDiet has been shown to decrease levels of low-density lipoprotein (LDL) cholesterol, triglycerides, and systemic inflammatory markers such as C-reactive protein (CRP), while increasing high-density lipoprotein (HDL) cholesterol. Additionally, its impact on reducing systolic and diastolic blood pressure further highlights its role in mitigating CVD risk^43,44^.

The microbiome-mediated effects of these diets are particularly noteworthy. SCFAs produced through the fermentation of dietary fiber enhance gut barrier integrity and reduce systemic endotoxemia, a key driver of atherosclerosis. In contrast, Western-style diets, rich in saturated fats and refined sugars, promote dysbiosis, which exacerbates systemic inflammation and lipid dysregulation^41,42^.

Integrating these dietary recommendations into clinical practice is essential for effective CVD prevention. The flexibility of the Mediterranean diet allows for its adaptation across different populations, making it a practical and sustainable approach to improving cardiovascular health. For instance, modifications such as the inclusion of local foods and variations in protein sources ensure cultural and regional applicability. Plant-based diets also offer a cost-effective strategy, particularly in resource-limited settings, where access to pharmacological therapies may be constrained^43,44^.

In conclusion, dietary interventions like the Mediterranean and plant-based diets offer substantial cardioprotective benefits by modulating the gut microbiome, reducing systemic inflammation, and improving metabolic health. As evidence continues to mount, these diets should be prioritized as foundational strategies in the prevention and management of CVD.

#### Fecal microbiota transplantation (FMT)

Fecal microbiota transplantation (FMT), the transfer of stool from a healthy donor to a recipient, is emerging as a therapeutic strategy to modulate gut microbiota composition. Initially developed to treat recurrent *Clostridium difficile* infections, FMT has shown promise in addressing metabolic and cardiovascular disorders associated with gut dysbiosis. Its mechanisms and preliminary evidence suggest significant potential for improving cardiovascular health^45,46^.

FMT restores gut microbial diversity and functionality by introducing a balanced microbiota into the dysbiotic intestinal environment. This intervention can enhance the production of beneficial metabolites such as short-chain fatty acids (SCFAs) and bile acids while reducing harmful byproducts like trimethylamine N-oxide (TMAO), which are implicated in atherosclerosis and systemic inflammation. Studies have shown that FMT can rebalance the Firmicutes/Bacteroidetes ratio and promote the growth of species associated with anti-inflammatory and cardioprotective effects^46,47^.

A notable pathway influenced by FMT is bile acid metabolism. Gut microbiota-modulated bile acids regulate metabolic health *via* receptors such as farnesoid X receptor (FXR) and G-protein-coupled bile acid receptor (TGR5). FMT has been shown to enhance bile acid deconjugation and improve bile acid profiles, fostering a more favorable cardiovascular metabolic environment^47^.

Emerging research highlights the impact of FMT on cardiovascular risk factors. In experimental autoimmune myocarditis models, FMT reduced myocardial inflammation and improved cardiac function by rebalancing gut microbial composition and decreasing pro-inflammatory cytokines^46^. Human studies also suggest FMT may improve cardiometabolic parameters such as lipid profiles and insulin sensitivity. For instance, a systematic review and meta-analysis revealed significant improvements in high-density lipoprotein (HDL) levels and reductions in insulin resistance following FMT interventions^48^.

Although the direct effect of FMT on cardiovascular events remains under investigation, these findings suggest that addressing dysbiosis can attenuate systemic inflammation and metabolic imbalances that predispose individuals to cardiovascular disease^45^. Some studies show improved lipid metabolism and insulin sensitivity post-FMT, while others report no significant changes in blood pressure or systemic inflammation^45^. A randomized controlled trial in obese patients with metabolic syndrome found that FMT improved insulin sensitivity but did not significantly alter lipid profiles or blood pressure^47^. Differences in outcomes may stem from variability in donor microbiota composition, host microbiome receptivity, and long-term sustainability of gut microbiota changes^48^.

Despite its potential, FMT faces several challenges. Ethical and logistical considerations, including donor selection, screening for pathogens, and standardization of procedures, remain unresolved. Long-term safety data are limited, and potential risks, such as pathogen transmission and immune reactions, require careful evaluation. Additionally, individual variations in response to FMT highlight the need for personalized approaches to maximize efficacy^46,48^.

In conclusion, FMT represents a novel therapeutic avenue for modulating gut microbiota and improving cardiovascular outcomes. Further clinical trials are needed to validate its efficacy, optimize protocols, and elucidate its long-term benefits and risks.

#### Pharmacological modulation of gut metabolites

Pharmacological strategies targeting gut metabolites have emerged as innovative approaches to mitigate cardiovascular disease (CVD) risk. Among these, modulation of trimethylamine-N-oxide (TMAO) pathways, gut-derived bile acids, and other microbial metabolites have shown promise in reducing systemic inflammation, atherogenesis, and metabolic imbalances linked to CVD^49,50^.

TMAO, a proatherogenic metabolite derived from the microbial metabolism of dietary choline and L-carnitine, is a key target for pharmacological interventions. Inhibitors that block TMA synthesis by targeting microbial enzymes such as choline TMA lyase (CutC/D) and carnitine Rieske-type monooxygenase (CntA/B) have demonstrated efficacy in reducing TMAO levels and attenuating atherosclerosis in animal models^51,52^. For example, polyphenol-rich compounds, such as those derived from tomato extracts, have been shown to remodel gut microbiota and decrease TMAO production, providing both metabolic and cardiovascular benefits^51^.

Additionally, ongoing clinical trials are exploring small-molecule inhibitors designed to selectively inhibit TMAO synthesis without disrupting the broader gut microbiome. These agents aim to reduce thrombosis potential, vascular inflammation, and foam cell formation, all of which are mediated by TMAO^52^.

Gut microbiota-mediated bile acid metabolism represents another therapeutic target. Bile acids, which interact with nuclear receptors such as the farnesoid X receptor (FXR) and G-protein-coupled bile acid receptor (TGR5), influence cholesterol metabolism, systemic inflammation, and insulin sensitivity. Pharmacological interventions aimed at modifying bile acid composition—either through microbial enzyme inhibition or synthetic bile acid analogs—have shown promise in preclinical studies. These strategies improve lipid profiles, enhance glucose metabolism, and mitigate CVD progression^49,50^.

Beyond TMAO and bile acids, other microbial metabolites such as short-chain fatty acids (SCFAs) and aromatic amino acid-derived compounds (e.g., phenylacetylglutamine) are being investigated for their roles in CVD. SCFAs, produced by fiber-fermenting bacteria, exhibit anti-inflammatory properties and regulate blood pressure. Drugs that enhance SCFA production or mimic their effects are under development. Similarly, inhibitors targeting pathways associated with aromatic amino acid metabolism, such as indoxyl sulfate and p-cresol sulfate, aim to reduce their proinflammatory and prothrombotic effects, thereby lowering CVD risk^50,52^.

While these strategies hold great promise, several challenges must be addressed. Ensuring specificity to target harmful metabolites without disrupting beneficial microbial functions is critical. Additionally, individual variability in microbiota composition necessitates a personalized approach to maximize efficacy. Further clinical trials are required to validate these therapies and assess their long-term safety and effectiveness in diverse populations^49,51^.

In conclusion, pharmacological modulation of gut metabolites offers a novel pathway to manage and reduce cardiovascular risk. With ongoing advancements in microbiome science, these strategies may soon complement or even replace traditional therapies for CVD.

#### Microbiome-based precision medicine

The integration of microbiome science into precision medicine is revolutionizing approaches to cardiovascular disease (CVD) management. The gut microbiota’s role in modulating metabolic and inflammatory pathways positions it as a critical factor in individualized therapeutic strategies. By leveraging microbiome profiling and advanced ‘omics’ technologies, clinicians can tailor interventions to address the unique microbiota composition of each patient, thereby optimizing cardiovascular outcomes^53,54^.

Microbiome profiling, enabled by next-generation sequencing (NGS) technologies, provides a detailed understanding of an individual’s microbial community structure and function. Techniques such as metagenomics and metabolomics allow for the identification of dysbiotic patterns associated with CVD, including shifts in short-chain fatty acid (SCFA) production, trimethylamine-N-oxide (TMAO) levels, and inflammatory markers^55^. These profiles inform personalized therapeutic strategies, ranging from dietary modifications to targeted microbial therapies.

For example, microbiome sequencing has revealed significant inter-individual variability in the gut microbiota’s capacity to metabolize dietary nutrients, influencing CVD risk. Patients with microbiota profiles that favor high TMAO production may benefit from dietary interventions to limit choline and carnitine intake, alongside potential use of TMAO inhibitors^53^. Similarly, profiling can identify specific microbial taxa or pathways to target with prebiotics, probiotics, or fecal microbiota transplantation (FMT)^54^. Microbiota profiling in patients with high TMAO production led to personalized dietary recommendations that reduced TMAO levels by 35%^55^.

Recent studies demonstrate the potential of microbiome-targeted therapies in precision medicine. For instance, microbial pathways have been implicated in modulating host lipid metabolism and systemic inflammation, suggesting that interventions targeting these pathways could mitigate CVD progression^54^. Personalized microbiome-based approaches, such as CRISPR-Cas-mediated editing of harmful microbial genes, are being explored as advanced therapeutic options^55^. Pilot trials using CRISPR-Cas editing of gut microbiota genes have shown potential in selectively removing harmful bacteria associated with metabolic dysregulation^54^.

Furthermore, the integration of microbiome data with other ‘omics’ datasets, including genomics and proteomics, enhances predictive accuracy for CVD risk stratification and therapeutic response. For example, pharmacogenomics-guided therapy for hyperlipidemia, informed by microbiome interactions, can optimize the use of lipid-lowering agents such as statins and PCSK9 inhibitors^54^. Pharmacogenomics-informed statin therapy improved lipid-lowering efficacy and reduced drug intolerance^53^.

Despite its promise, the implementation of microbiome-based precision medicine faces challenges. Variability in microbiome composition due to environmental factors, diet, and genetics necessitates robust standardization of profiling techniques and data interpretation. Additionally, the causal relationships between specific microbiome alterations and CVD remain complex and require further elucidation through longitudinal and mechanistic studies^53^.

Future research should focus on expanding microbiome-wide association studies (MWAS) and developing cost-effective, scalable diagnostic tools. The integration of artificial intelligence and machine learning in microbiome analysis holds potential for real-time, actionable insights. Advances in this field could enable routine microbiome assessment as part of personalized CVD management protocols^55^.

In conclusion, microbiome-based precision medicine represents a transformative approach to CVD management, emphasizing individualized care through targeted modulation of the gut microbiota. As research advances, these strategies may become integral to preventing and treating cardiovascular diseases.

## Future directions

The rapidly evolving field of microbiome research presents numerous opportunities to advance cardiovascular disease (CVD) management. However, several gaps remain that warrant further investigation. First, the long-term safety and efficacy of microbiome-targeted interventions, including fecal microbiota transplantation (FMT) and TMAO inhibitors, must be validated through large-scale, multicenter clinical trials. These studies should address diverse populations to account for variability in microbiome composition and patient response.

Second, the development of microbiome-based biomarkers for early detection and risk stratification of CVD represents a promising avenue. Advances in next-generation sequencing and multi-omics technologies could facilitate the identification of microbial signatures associated with cardiovascular health and disease progression. Integrating these biomarkers into clinical practice could enable personalized therapeutic strategies, optimizing outcomes while minimizing adverse effects.

Third, emerging technologies such as CRISPR-Cas systems for precision editing of the microbiome offer potential for targeted manipulation of pathogenic microbial pathways. These approaches, alongside advanced computational tools and artificial intelligence could revolutionize microbiome-based precision medicine by providing tailored interventions based on an individual’s unique microbiota composition.

Finally, translating microbiome science into accessible and cost-effective therapies remains a critical challenge. Future efforts should prioritize the scalability of interventions like probiotics, prebiotics, and dietary programs while ensuring equitable access across different populations. Addressing these challenges will require interdisciplinary collaboration between microbiologists, clinicians, and policymakers.

In summary, while the gut microbiome presents a novel frontier in CVD management, substantial efforts are needed to overcome current limitations and translate scientific insights into practical clinical applications. The continued evolution of this field holds the potential to redefine cardiovascular care, paving the way for more personalized, effective, and sustainable therapeutic strategies.

## Conclusion

The interplay between the gut microbiome and cardiovascular disease (CVD) represents a rapidly evolving field with significant clinical implications. This review highlights the role of microbiome dysbiosis in driving systemic inflammation, metabolic dysfunction, and vascular pathology. Beyond confirming known mechanisms such as TMAO production and gut barrier dysfunction, emerging research underscores the potential of microbiome-based precision medicine, including personalized dietary interventions, targeted probiotics, and pharmacological modulation of microbial metabolites. While microbiome-targeted strategies offer a promising adjunct to traditional CVD treatments, their clinical translation remains hindered by interindividual variability and a need for standardized therapeutic protocols. Future research should focus on integrating microbiome profiling into cardiovascular risk assessment, optimizing interventions for sustained efficacy, and addressing regulatory challenges. Advancing our understanding of the gut-heart axis could pave the way for microbiome-based therapies that complement conventional approaches, ultimately improving cardiovascular outcomes and patient care.
